# Mechanisms for Improving Hepatic Glucolipid Metabolism by Cinnamic Acid and Cinnamic Aldehyde: An Insight Provided by Multi-Omics

**DOI:** 10.3389/fnut.2021.794841

**Published:** 2022-01-11

**Authors:** You Wu, Ming-hui Wang, Tao Yang, Tian-yu Qin, Ling-ling Qin, Yao-mu Hu, Cheng-fei Zhang, Bo-ju Sun, Lei Ding, Li-li Wu, Tong-hua Liu

**Affiliations:** ^1^Key Laboratory of Health Cultivation of the Ministry of Education, Beijing University of Chinese Medicine, Beijing, China; ^2^Key Laboratory of Health Cultivation of Beijing, Beijing University of Chinese Medicine, Beijing, China; ^3^Department of Science and Technology, Beijing University of Chinese Medicine, Beijing, China

**Keywords:** cinnamic acid, cinnamaldehyde, liver, transcriptome, proteome, glucolipid metabolism, db/db

## Abstract

Cinnamic acid (AC) and cinnamic aldehyde (AL) are two chemicals enriched in cinnamon and have been previously proved to improve glucolipid metabolism, thus ameliorating metabolic disorders. In this study, we employed transcriptomes and proteomes on AC and AL treated db/db mice in order to explore the underlying mechanisms for their effects. Db/db mice were divided into three groups: the control group, AC group and AL group. Gender- and age-matched *wt*/*wt* mice were used as a normal group. After 4 weeks of treatments, mice were sacrificed, and liver tissues were used for further analyses. Functional enrichment of differentially expressed genes (DEGs) and differentially expressed proteins (DEPs) were performed using Gene Ontology (GO) and Kyoto Encyclopedia of Genes and Genomes (KEGG) databases. DEPs were further verified by parallel reaction monitoring (PRM). The results suggested that AC and AL share similar mechanisms, and they may improve glucolipid metabolism by improving mitochondrial functions, decreasing serotonin contents and upregulating autophagy mediated lipid clearance. This study provides an insight into the molecular mechanisms of AC and AL on hepatic transcriptomes and proteomes in disrupted metabolic situations and lays a foundation for future experiments.

## Introduction

The global prevalence of metabolic diseases, including diabetes and obesity, along with that of non-alcoholic fatty liver disease and dyslipidemia, has risen in recent years (1–3). As a result, an economic burden on patients and the healthcare system has occurred, making these diseases major threats to public health (4, 5). Caused by similar pathogenesis, these metabolic diseases are highly related to each other, and a common pathological change in these diseases is the disruption in glucose, lipid and energy metabolism (6, 7).

The occurrence of glucolipid metabolism could be attributed to many pathogeneses. Insulin resistance, inflammation, mitochondrial disfunction, autophagy disruption as well as gut microbiota reconstruction may all lead to metabolic disorders. As an essential metabolic organ, the liver plays a central role in the regulation of carbohydrate and lipid metabolism, thus maintains the internal energy balance (8–10).

The ongoing epidemic of metabolic diseases indicates the current prevention and treatment approaches for metabolic diseases are not successful in the long term (11). Thus, finding new strategies to prevent and treat glucolipid metabolic disorder is becoming an important research subject. Dietary change is considered to be one of the ideal treatments for obesity and is recommended by the National Heart, Lung, and Blood Institute (12). Dietary supplements and natural products are usually highly safe, and many of them are proven to be effective in treating metabolic diseases (13–15), among which cinnamon (mainly *Cinnamomum verum* J.S. Presl and *C. cassium* Blume from *Cinnamomum* genus) and its components were demonstrated to possess multiple therapeutic effects, including anti-diabetes, reducing body weight, hypolipidemia and enhancing insulin sensitivity (16–20). Hence, using dietary supplements and chemicals derived from cinnamon on a glycemic and lipidemic control seems promising.

**Figure 1:**
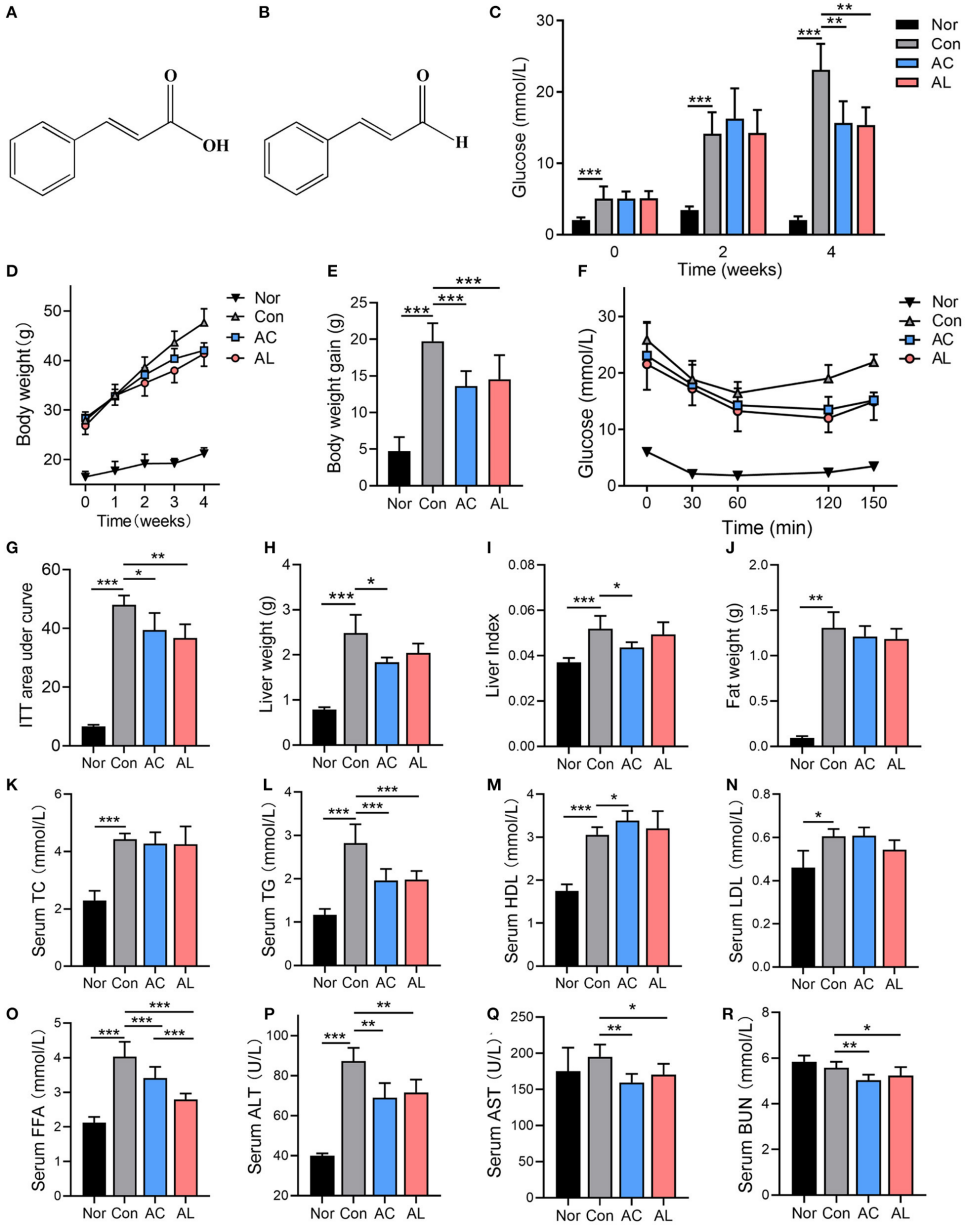
Effects of AC and AL on glucolipid metabolism in mice. **(A,B)** The chemical structure of AC **(A)** and AL **(B)**. **(C,D)** Fast glucose levels **(C)** and body weight **(D)** of mice. **(E)** Body weight gain of mice at the fourth week of treatment. **(F)** Glucose levels of mice during ITT (hypodermically injected with insulin 1 IU/kg body weight). **(G)** Area under curve of ITT experiment. **(H–J)** Liver weight, liver index and epididymal fat weight of mice, respectively. **(K–R)** Serum lipid profile and ALT, AST and BUN of mice measured by automated chemistry analyzer. Nor: *wt*/*wt* mice treated with vehicle. Con: db/db mice treated with vehicle. AC: db/db mice treated with 20 mg/kg bodyweight/day cinnamic acid. AL: db/db mice treated with 20 mg/kg bodyweight/day cinnamic aldehyde. One-way ANOVA analysis applied for statistical analysis, *N* = 6–7/group. ^*^p < 0.05, ^**^p < 0.01, ^***^p < 0.001.

The *Cinnamomum* genus encompasses about 250 species, and several of them have been used as spices for centuries (21, 22). Cinnamon used in seasoning material consists of dried inner bark of trees from the genus. Cinnamic acid (AC) and cinnamic aldehyde (also known as cinnamaldehyde, AL) are two important chemicals found in cinnamon (Figures 1A,B) (23). Previous studies revealed that cinnamic acid could improve lipid metabolism and reduce lipid overaccumulation (24–26) as well as regulate glucose metabolism and ameliorate hyperglycemia (27, 28). Cinnamic aldehyde is also widely investigated and was proved to be effective in improving insulin sensitivity and boosting mitochondrial functions (29, 30). Furthermore, previous studies reported that AL is unstable when administered to animals, and a major part of the absorbed AL is converted to AC in the body (31, 32). Thus, it is plausible to assume that the mechanisms of effects of these two chemicals share some similarities. Although the effects of both chemicals have been confirmed by many *in vitro* and *in vivo* studies, the underlying mechanisms remain poorly understood and still need elucidation.

Compared with traditional investigation approaches, omics such as genome, transcriptome, proteome and metabolome studies interpret different levels of molecular changes, which provide more comprehensive understanding of potential mechanisms of drugs and diseases. Multi-omics analysis is an emerging trend for systematically examining pharmacological mechanisms in medical research (33). Hence, in this study we applied transcriptomes and data-independent acquisition (DIA) proteomes on liver samples of db/db mice treated with AC or AL and performed integrated analysis in order to produce a comprehensive perception of their pharmacological effects and a foundation for future studies on these two chemicals.

## Materials and Methods

### Animal Experiments

All animal experiments performed in this study were approved by the Animal Care and Ethics Committee of Beijing University of Traditional Chinese Medicine (approval code: No. BUCM-4-2019031002-1088). Mice were purchased form Nanjing Biomedical Research Institute of Nanjing University (Nanjing, China). AC (No. S30461, ≥99.5% purity) and AL (No. 24035, ≥95% purity) were purchased from Shanghai YuanYe Bio-Technology Co., Ltd (Shanghai, China). Chemicals were dissolved in 0.5% carboxymethyl cellulose buffer (Solarbio Science & Technology, Beijing, China) to a 0.2% concentration and thoroughly stirred for 30 min on a magnetic stirrer every time before administered to mice by gavage.

21 five-week-old male C57BL/KsJ-db/db mice and 7 C57BL/KsJ-*wt*/*wt* mice of the same gender and age were maintained in a specific-pathogen-free facility under 12/12 h light/dark cycles and fed on a normal chow diet (Lot: SPF-F02, SPF Biotechnology, Beijing, China) and water *ad libitum* throughout the study. Following a 1-week acclimation, db/db mice were randomly divided into 3 groups with 7 mice in each group: the cinnamic acid group (acid, treated with AC 20 mg/kg body weight every day), cinnamic aldehyde group (aldehyde, treated with AL 20 mg/kg body weight every day), control group (con, treated with vehicle every day). *Wt*/*wt* mice were used as normal group (nor, treated with vehicle every day). Treatments lasted 4 weeks, and the body weight of mice was measured every week and food intake was recorded every three days. Blood glucose was measured at the beginning and middle of the treatment after overnight fasting. Blood samples were collected from tail vein and measured by a portable glucometer (Glucocard 01-mini, Arkray, Kyoto, Japan).

### Insulin Tolerance Test (ITT)

ITT was performed after 4 weeks of treatment. Mice were fasted for 4 h and hypodermically injected with insulin (Novolin R) 1 IU/kg body weight. Glucose levels were measured at 0, 30, 60, 120, and 150 min after injection using blood drawn from tail vein by a portable glucometer. Area under curve (AUC) of ITT was calculated as follows:


AUC=0.25×G0+0.5×G30+0.75×G60+0.75×G120+0.25×G150

(G0, G30, G60, G120, and G150 represent blood glucose at 0, 30, 60, 120, and 150 min, respectively).

### Biochemical Measurements

Mice were sacrificed 3 days after ITT experiment, and serum samples were collected and centrifuged at 3,000 rpm for 15 min at 4°C in order to get the serum. 200 μl of serum from each sample were loaded onto an automated chemistry analyzer (AU480, Beckman Coulter, Brea, CA, USA) and the blood glucose levels, total cholesterol (TC), triglyceride (TG), high-density lipoprotein (HDL), low-density lipoprotein (LDL), alanine transaminase (ALT), aspartate aminotransferase (AST), free fatty acid (FFA) and blood urea nitrogen (BUN) were measured. Serum from all mice were included in the biochemical measurements (*N* = 7 per group). Furthermore, the hepatic ATP content (No. A095-1-1, Nanjing Jiancheng Bioengineering, Nanjing, China) and serotonin content (Cat. CSB-E08365m, Cusabio Technology, Wuhan, China) were tested using commercial kits according to the manufacturers' instructions. The ATP was measured using the colorimetric method, and the serotonin content was measured by enzyme linked immunosorbent assay (ELISA) (*N* = 6 per group).

### Morphological Assessment

Tissues were harvested and weighed on a precision balance (Mettler-Toledo, Columbus, OH, USA). Epidydimal fat from only the left side of the mice was weighed. Liver and adipose tissues were cut into small cubes, one piece of each tissue was fixed in 4% paraformaldehyde solution for 48 h then embedded in paraffin and cut into slices, the remaining tissues were harvested and frozen with liquid nitrogen before preserved in −80°C. Embedded tissues were cut into 4-μm-thick slices using a microtome for further assessments. Hematoxylin and eosin (H&E) staining was performed on fixed livers and epidydimal adipose tissues for morphological observation. In addition, periodic acid–Schiff (PAS) staining was conducted on liver tissues to assess hepatic glycogen content. For quantitative assay, the hepatic steatosis degree was scored as previously described (34) and the adipocyte size was measured using Image J software (NIH Image, Bethesda, MD, USA).

### RNA Extraction and Library Construction

Liver tissues from 4 mice in each group were used for RNA extraction. Total RNA was extracted using a commercial kit (*mir*Vana miRNA Isolation Kit, Ambion, Austin, TX, USA) according to the manufacturer's protocol. The experiments followed the procedure of extracting total RNAs with this kit. RNA quality and integrity were evaluated using an Agilent 2100 Bioanalyzer (Agilent Technologies, Santa Clara, CA, USA). Qualified RNA (RNA integrity number ≥7) was used for library construction. Libraries were constructed using a TruSeq Stranded mRNA LTSample Prep Kit (Illumina, San Diego, CA, USA) according to instructions.

### RNA Sequencing and Differentially Expressed Gene (DEG) Analysis

Libraries prepared above were then loaded on the Illumina HiSeq^TM^ X Ten platform to generate raw reads. After impurity removal, clean data were mapped to *Mus musculus* (GRCm38.p6) using HISAT (35). Read counts of genes were acquired by HTSeq-count (36), and expression levels were calculated using the fragments per kb per million reads (FPKM) method (37). The expression levels were standardized and analyzed using the DESeq (version 3.12) R package. DEGs were determined as those with a *p*-value of <0.05 and fold change of ≥1.5.

### Real-Time Quantitative PCR (RT-qPCR)

Total RNA was extracted as described in Section RNA Extraction and Library Construction. Reverse transcription was performed using a commercial kit (Lot: AT341-92, TransGen Biotech, Beijing, China) according to instructions. 1 μl obtained cDNA was mixed with 5 μl PerfectStart™ Green qPCR SuperMix (Lot:AQ601, TransGen Biotech), 0.2 μl forward primer, 0.2 μl reverse primer and 3.6 μl nuclease-free water to form a 10 μl system before loaded onto a qPCR instrument. Real-time PCR was performed on LightCycler^®^ 480 II Real-time PCR Instrument (Roche Molecular Systems, Inc., Swiss) in a 384-well optical plate at 94°C for 30 s, followed by 45 cycles of 94°C for 5 s, 60°C for 30 s. The mRNA expression levels were normalized to β-actin and calculated by 2^−ΔΔCt^ method. Primers used in qPCR are listed in Supplementary Table 1.

### Protein Extraction and Preparing

Liver tissues from 4 mice in each group were used for protein extraction. Liver tissues went through liquid nitrogen grinding and were suspended in 300 μl lysis buffer containing 1 mM phenylmethanesulfonyl fluoride and sonicated on ice. Solutions were centrifuged at 12,000 × g for 10 min at room temperature twice to acquire the total protein. The concentration of protein solution was measured using the BCA method, and the quality was tested by SDS-PAGE with G250 as described previously (38). Fifty micrograms of protein from each sample were incubated with sequencing-grade trypsin (1 μg/μL, Hualishi Scientific, Beijing, China) for enzymolysis, and the digested peptides were desalted using a C18-Reverse-Phase SPE Column. The column was washed with methanol, 0.1% trifluoroacetic acid (TFA)/90% acetonitrile and 0.1% TFA/water. Then, the samples were loaded on the column 3 times, and 0.1% TFA/water was used to wash the column 3 times. Finally, the peptides were eluted with 0.1% TFA/90% acetonitrile 3 times and lyophilized for further DIA and PRM examination.

### LC-MS/MS Analysis and Differentially Expressed Protein (DEP) Analysis

Reversed-phase separation was performed using an Agilent Zorbax Extend–C18 column (2.1 × 150 mm, 5 μm) on a 1100 HPLC System (Agilent, Santa Clara, CA, USA). Separated peptides were lyophilized for mass spectrometry. A Q-Extractive HF mass spectrometer (Thermo Fisher Scientific, Bremen, Germany) was used for mass spectrometry analysis.

For DIA mass spectrum scanning, full MS scans were acquired in the scan range of 350–1,250 m/z and maximum injection time of 100 ms with a mass resolution of 120,000 and AGC target value set at 3e6. For MS2, scanning was performed with a resolution of 30,000 and isolation window set to 26 m/z. Acquired DIA spectra were matched with a reference library generated by traditional data-dependent acquisition (DDA) approaches. Raw data from DDA analysis were processed and compared to the UniProt database using Spectronaut Pulsar software (Biogenosys, Schlieren, Switzerland). DEPs were determined as those with a *p*-value of <0.05 and fold change of ≥1.2.

### Bioinformatics Analysis

Principal component analysis (PCA) was conducted to determine the relationships between samples in each group. Gene set enrichment analysis (GSEA) (39) was performed using GSEA 4.1.0 software to analyze the transcriptomic data from db/db mice. Genes were ranked according to their expression in two groups using Signal2Noise as a metric for ranking genes. Gene Ontology (GO) and Kyoto Encyclopedia of Genes and Genomes (KEGG) were used as gene set databases for the GSEA analysis (Supplementary Table 2). The relationship between mRNA expression and protein expression of identified key factors was visualized by a four-quadrant diagram (Supplementary Figure 1).

Functional annotation and pathway analysis of DEGs and DEPs employed the GO database and the KEGG database. A GO double-donut chart was generated to evaluate the overlap between DEGa and DEPs.

### Parallel Reaction Monitoring (PRM) Assay

The DIA proteome results were further validated by the PRM assay, which works as a protein validation approach that acquires full MS/MS spectra with higher specificity and accuracy in protein validation than the traditional antibody-based method (40, 41). For PRM validation of selected proteins, protein was extracted from liver samples and enzymolized and desalted as described in Section RNA Sequencing and Differentially Expressed Gene (DEG) Analysis, and a 10X iRT standard peptide mix (prepared according to the user manual) was added to desalted peptide samples. A list of unique peptides from DDA analysis was prepared (2–5 per protein, Supplementary Table 3). The peptides were separated at flow rate of 300 nL/min using a 75 μm × 15 cm, nanoViper, C18, 3 μm, 100Ȧ column (Acclaim, PepMap). The gradient was as follows: 0–82 min, 5–44% B, 82–84 min, 44–90% B, 84–90 min, 90–100% B (buffer A: HPLC H_2_O, 0.1% formic acid, buffer B: 80% ACN, 0.1% formic acid). Then, the peptides were transferred into a gaseous phase, and mass chromatography was conducted using a Q Exactive HF mass spectrometer (Thermo Fisher Scientific). The MS mood was measured at 70,000 resolution, and MS/MS spectra were acquired at 15,000 resolution (at 1.2 m/z isolation window width) with the AGC target set to 2e5. The spectra were further analyzed with Skyline (42).

### Statistical Analysis

Results were analyzed using SPSS 23.0 software (SPSS, Chicago, IL, USA), and all data are presented as mean ± standard deviation (SD). For protein expression data from DIA experiment, unpaired two-tailed Student's *t-*tests were applied to determine the *p*-value of expression and identify DEPs between two groups. For data from more than two groups, analysis was run under one-way ANOVA with Dunnett's post-test (unequal variance) or least significant difference post-test (equal variance). Statistically significant differences were defined as *p* < 0.05 (^^*^^p < 0.05, ^^**^^p < 0.01, ^^***^^p < 0.001).

## Results

### Effects of AC and AL on Glucolipid Metabolism in Mice

Oral administration of AC and AL did not affect the appetite of db/db mice, as suggested by the food intake data (Supplementary Figure 2a). Db/db mice in the control group displayed a significantly disrupted glucolipid metabolism state, as shown by the phenotypes, the blood glucose level and body weight, which were significantly higher compared to the normal group (Figures 1C,D). Compared with the control group, AC and AL significantly ameliorated hyperglycemia and obesity in db/db mice after 4 weeks of treatment. AC and AL reduced fast blood glucose levels at the end of treatment (Figure 1C). Two chemicals both significantly suppressed body weight gain in db/db mice (Figures 1D,E). The ITT experiment at the fourth week of the treatment showed evaluated baseline blood glucose level in db/db mice, and the db/db mice treated with AC and AL were more sensitive to hypodermically injected insulin, as revealed by the AUC results (Figures 1F,G).

Meanwhile, AC treatment significantly reduced the liver weight of db/db mice and reduced liver index. AL also reduced the liver mass; however, the data failed to reach statistical significance (Figures 1H,I). Although the treatments showed a tendency to reduce the weight of epidydimal fat, the data were not statistically significant (Figure 1J). The control group showed elevated serum lipid content compared to wild type mice, and AC and AL partially alleviated these changes (Figures 1K–O). Although AC and AL treatments did not seem to have a noticeable impact on the serum TC levels of db/db mice, the TG in the AC and AL groups was significantly decreased (by 30.5 and 23.4%, respectively), and the FFA levels in the AC and AL groups were significantly lower than those of the control group (by 15.4 and 27.5%, respectively). AC treatment also significantly increased serum HDL levels. It is worth noting that the AST, ALT and BUN levels in treated groups were significantly reduced, suggesting that oral intake of AC or AL may ameliorate abnormal renal and liver function in db/db mice (Figures 1P–R).

### Histological Observation

**Figure 2:**
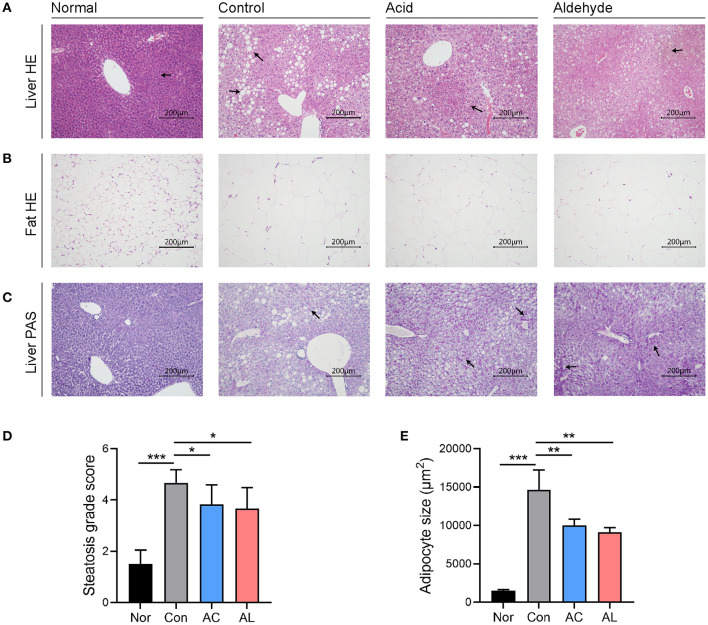
Histological observation. **(A)** HandE staining of liver tissues harvested from mice. **(B)** HandE staining of epididymal fat (the left side) of mice. **(C)** PAS staining of liver from mice. All images were recorded under 200× magnification. **(D)** Steatosis grade scores of liver HandE staining. **(E)** Adipocyte size measured from epidydimal fat HandE staining. (One-way ANOVA analysis applied for statistical analysis, *N* = 3/group.) Normal: *wt*/*wt* mice treated with vehicle. Control: db/db mice treated with vehicle. Acid: db/db mice treated with 20 mg/kg bodyweight/day cinnamic acid. Aldehyde: db/db mice treated with 20 mg/kg bodyweight/day cinnamic aldehyde. ^*^p < 0.05, ^**^p < 0.01, ^***^p < 0.001.

H&E staining of the control group mice showed severe hepatic steatosis occurred in the liver. AC and AL reduced the lipid accumulation and partially restored disrupted hepatic cord structure (Figure 2A). Quantitative analysis showed the steatosis grades of treated groups were significantly lower than that of the control group (Figure 2D). Consistent with the changes observed in epidydimal fat weight, the histological observation of epidydimal fat tissues suggested that, compared with those of normal group, the adipocytes of db/db mice showed a massive expansion of size, and AC and AL reduced the size of ballooned adipocytes (Figures 2B,E). PAS staining of liver indicates the hepatic glycogen content was increased after treatments (Figure 2C).

### Overview of Transcriptome and Proteome Differences Between Groups

**Figure 3:**
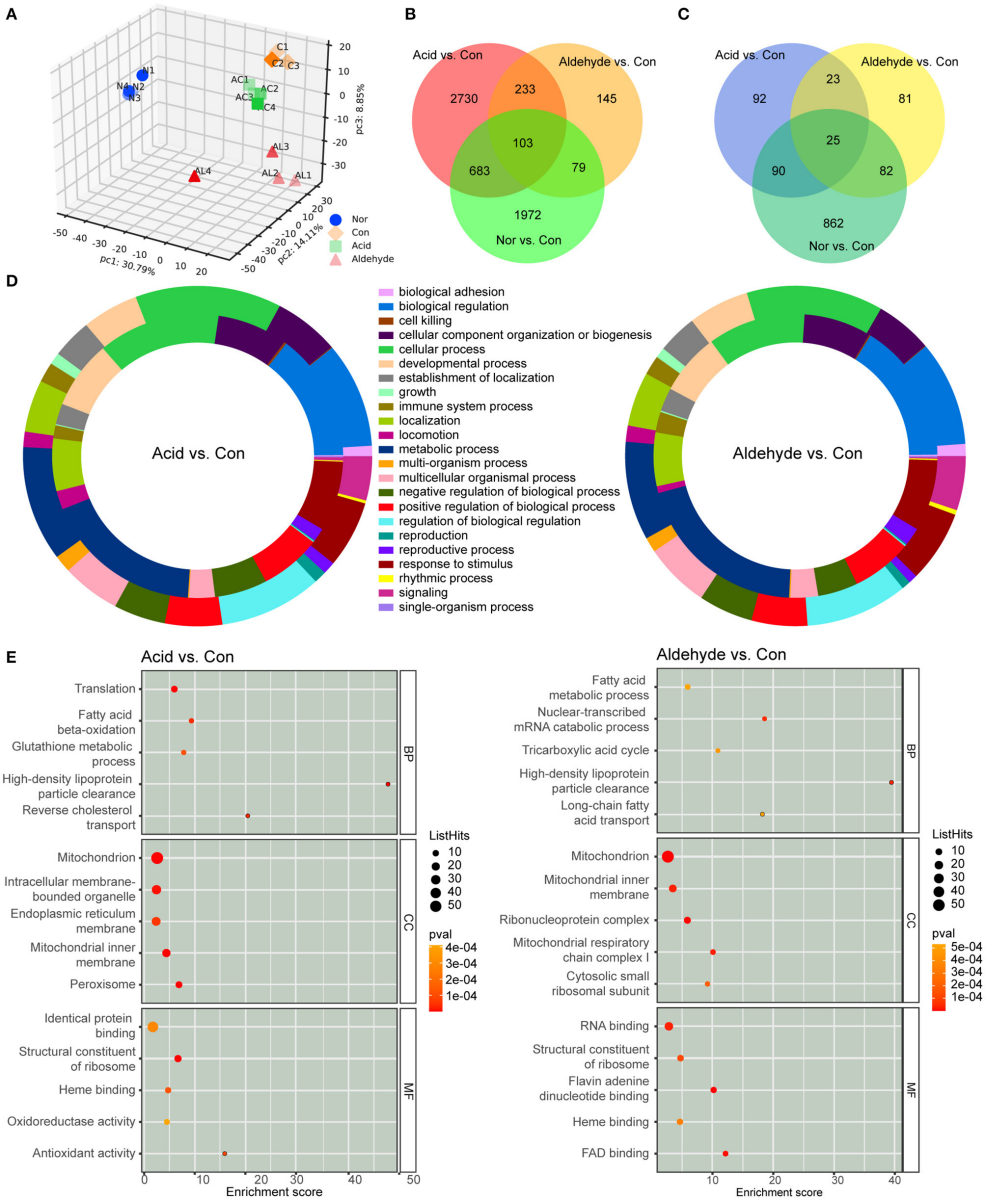
Overview of transcriptomes and proteomes of mice livers and functional enrichment of differentially expressed genes (DEGs) and differentially expressed proteins (DEPs). **(A)** Principal component analysis (PCA) of proteome results. **(B,C)** Venn diagram of DEGs **(B)** and DEPs **(C)**. **(D)** Donut diagram of GO enrichment of DEGs and DEPs between the acid group and control group and aldehyde group and control group. The outer ring indicates the functional annotation of DEGs, and the inner ring indicates the functional annotation of DEPs. **(E)** GO enrichment of DEPs depicted by bubble diagrams. The color indicates the *p*-value, and the size indicates the enrichment score of each pathway. Nor: *wt*/*wt* mice treated with vehicle. Con: db/db mice treated with vehicle. AC: db/db mice treated with 20 mg/kg bodyweight/day cinnamic acid. AL: db/db mice treated with 20 mg/kg bodyweight/day cinnamic aldehyde.

The effects of AC and AL on db/db mice were investigated by RNA-Seq analysis and DIA proteomic analysis. PCA analysis of the outcomes indicated a clear difference of the transcriptomes and proteomes between groups (Figure 3A and Supplementary Figure 2b). For the transcriptomic analysis, one sample was eliminated from the control group, normal group and AL group because of high deviation from others. A total number of 16,812 different protein-coding transcripts were identified from remained samples. Of these, 2,837 (about 16.9%) were significantly different between normal and control groups, suggesting the db/db mice were remarkably different to wild type mice in transcription levels. A total of 3,749 genes were significantly altered by ingestion of AC (2,201 were downregulated and 1,548 were upregulated) and 560 by AL (430 were down regulated and 130 were upregulated) when compared to the control group (Figure 3B).

For DIA proteomic analysis, 2,729 proteins were identified from all group sets. One sample was eliminated from the control group for the same reason as in the transcriptomic analysis. After elimination, samples were clearly divided into four groups in PCA analysis, showing a clear difference between groups and a relatively low heterogeneity inside groups. A total of 1,059 (about 38.8% of total identified proteins) DEPs were discovered between the normal group and control group; 230 proteins were significantly changed by AC treatment (109 were upregulated and 121 were downregulated) and 211 by AL treatment (116 were upregulated and 95 were downregulated) (Figure 3C).

### Functional Annotation and Enrichment Analysis of DEGs and DEPs

In order to identify the functions of DEGs and DEPs, GO enrichment analysis was performed. Functional annotation of DEGs and DEPs showed significant difference in glucolipid and energy metabolism pathways between db/db mice in the control group and *wt/wt* mice in the normal group. The donut diagram revealed that AC and AL significantly altered gene and protein expression in metabolic pathways. These two treatments showed a similar pattern in terms of molecular mechanisms, and a large portion of DEGs and DEPs are related to metabolic processes (Figure 3D). Indeed, KEGG enrichment of DEGs confirmed both chemicals regulated pathways related to metabolic processes, such as metabolism of lipids, amino acids and carbohydrates. Furthermore, a large number of DEGs are enriched in the endocrine system (Supplementary Figure 3). GO enrichment of DEPs showed that AC and AL especially regulated lipid metabolism including fatty acid metabolism, long-chain fatty acid and cholesterol transportation and HDL particle clearance (Figure 3E). It is noteworthy that a large number of the DEPs are enriched in cellular organelles such as mitochondria, which are essential for multiple metabolism pathways and the maintenance of energy balance.

### Effect of AC and AL on Key Factors Involved in Glucolipid Metabolism

**Figure 4:**
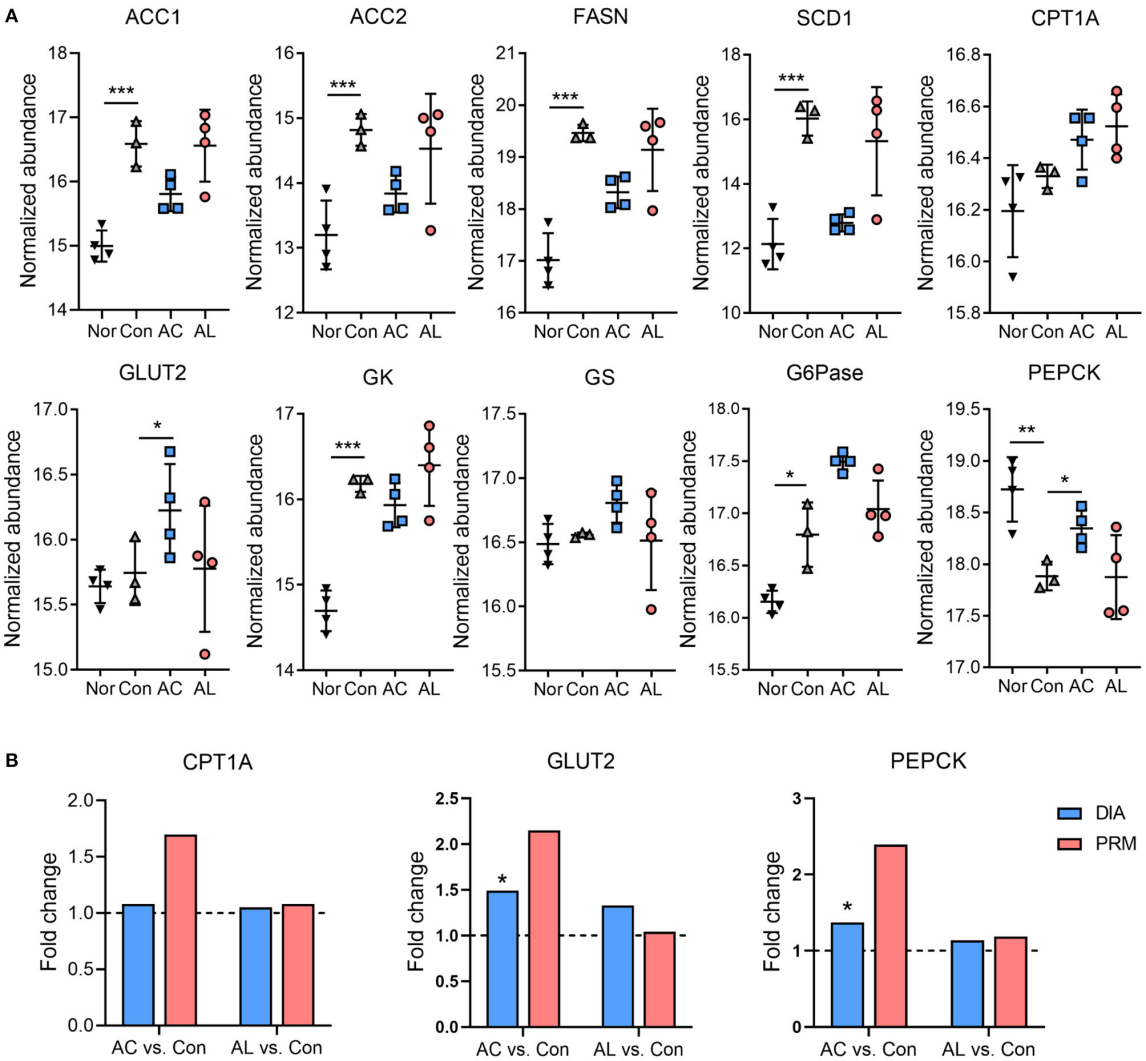
Effect of AC and AL on key factors involved in glucolipid metabolism. **(A)** Normalized abundance of indicated proteins in mice livers. Data are quantile normalized before log2 transformation. **(B)** Results of PRM analysis. Blue column indicates the fold change of DIA proteome analysis, and red column indicates the fold change of PRM verification. Nor: *wt*/*wt* mice treated with vehicle. Con: db/db mice treated with vehicle. AC: db/db mice treated with 20 mg/kg bodyweight/day cinnamic acid. AL: db/db mice treated with 20 mg/kg bodyweight/day cinnamic aldehyde. ^*^p < 0.05, ^**^p < 0.01, ^***^p < 0.001.

Several proteins involved in hepatic glucolipid metabolism were identified form proteome data, such as acetyl-CoA carboxylase (ACC), fatty acid synthase (FASN), stearoyl-CoA desaturase 1 (SCD1), carnitine palmitoyl transferase-1A (CPT1A), glucose transporter 2 (GLUT2), glucokinase (GK), glycogen synthase (GS), glucose-6-phosphatase (G6Pase) and phosphoenolpyruvate carboxykinase (PEPCK). Though non-significant, AC showed the tendency to inhibit the expression of lipogenesis enzymes, including ACC, FASN and SCD1, which is consistent with our previous findings (43). AC also significantly upregulated GLUT2. Compared with the control group, AC significantly increased the expression of PEPCK, and both chemicals showed no significant effect on G6Pase (Figure 4A). The expression of proteins from proteomic data were further confirmed by the PRM assay, as the results of PRM showed the same direction of change with the proteome (Figure 4B).

### Effect of AC and AL on Key Factors Involved in Glucolipid Metabolism

**Figure 5:**
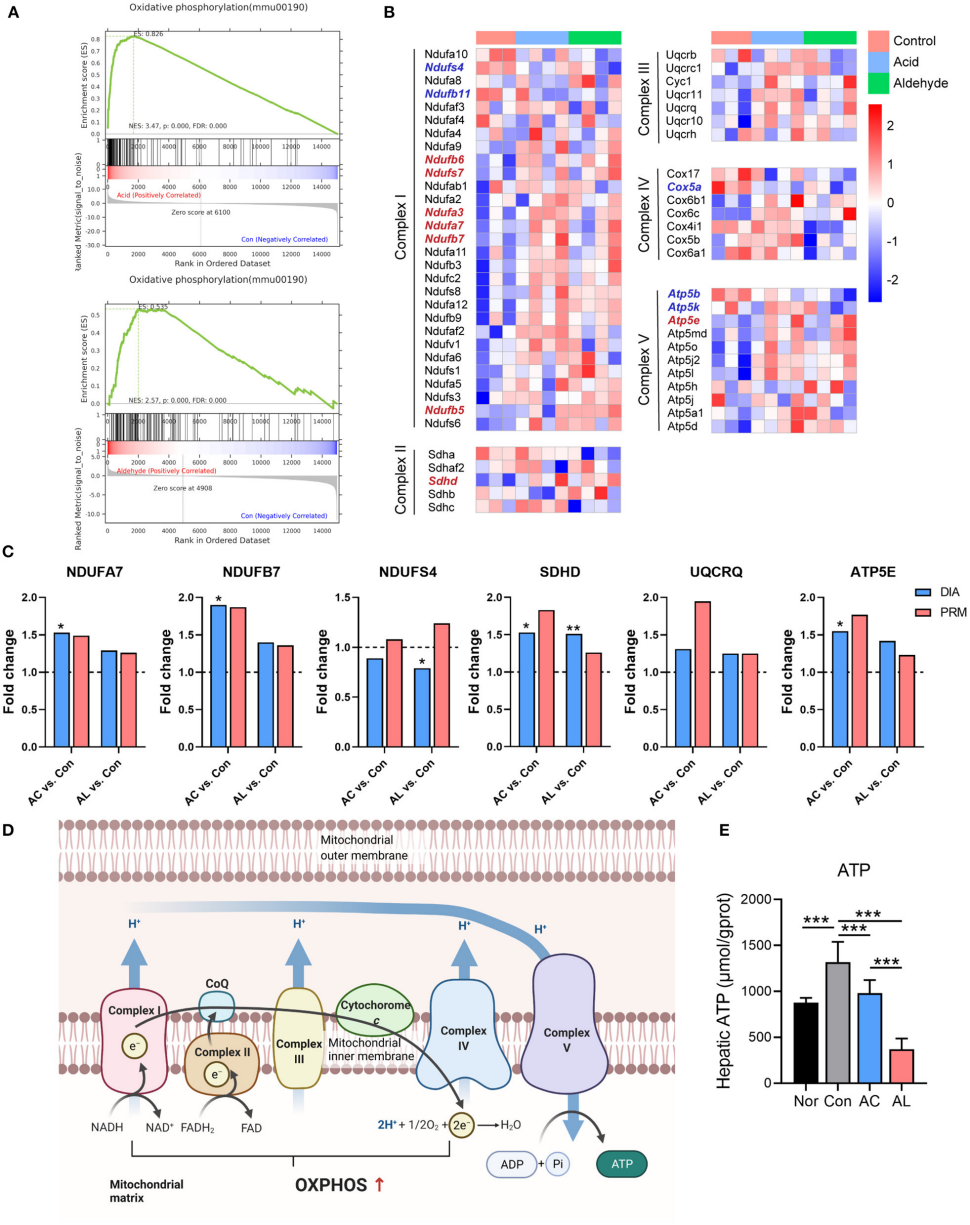
AC and AL improved mitochondrial function in db/db Mice. **(A)** Gene set enrichment analysis (GSEA) of oxidative phosphorylation (OXPHOS) pathway. **(B)** Heatmap of expression of proteins involved in OXPHOS pathway. Red indicates high expression level, and blue indicates low expression level. **(C)** Results from PRM analysis. Blue column indicates the fold change of DIA proteome analysis, and red column indicates the fold change of PRM verification. **(D)** Diagram of the OXPHOS pathway in mitochondria. **(E)** ATP content in mice livers tested by colorimetric method (one-way ANOVA analysis applied, *N* = 6/group). Nor: *wt*/*wt* mice treated with vehicle. Con/control: db/db mice treated with vehicle. AC/acid: db/db mice treated with 20 mg/kg bodyweight/day cinnamic acid. AL/aldehyde: db/db mice treated with 20 mg/kg bodyweight/day cinnamic aldehyde. ^*^p < 0.05, ^**^p < 0.01, ^***^p < 0.001.

**Figure 6:**
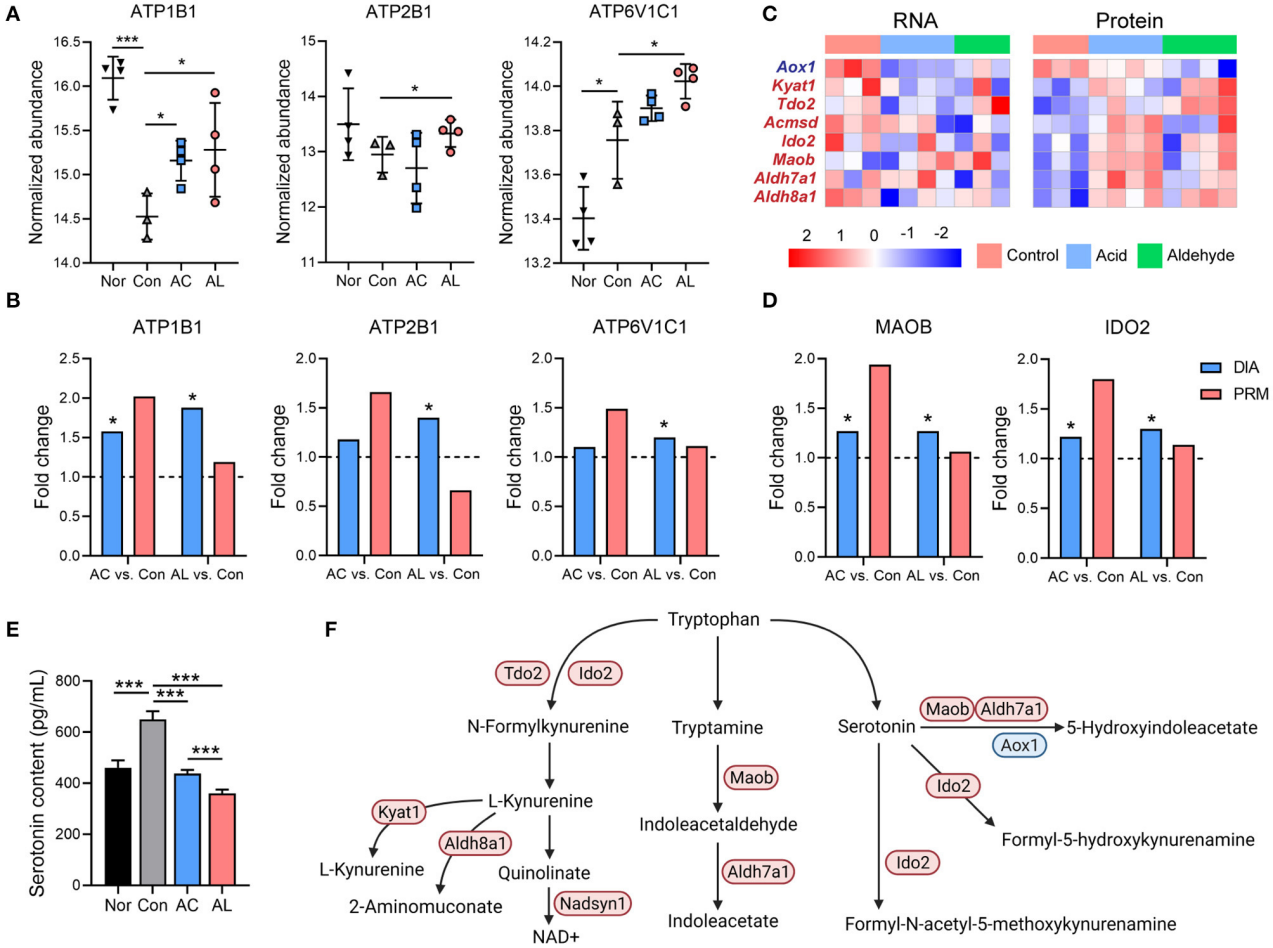
Effect of AC and AL on tryptophan metabolism in db/db mice livers. **(A)** Normalized abundance of indicated proteins in mice livers. Data are quantile normalized before log2 transformation. **(B,D)** Results from PRM analysis. Blue column indicates the fold change of DIA proteome analysis, and red column indicates the fold change of PRM verification. **(C)** Heatmap of expression of DEPs involved in tryptophan metabolism pathway. Red indicates high expression level, and blue indicates low expression level. **(E)** Serotonin content in mice livers tested by enzyme linked immunosorbent assay (ELISA) (one-way ANOVA analysis applied, *N* = 6/group). **(F)** Diagram of tryptophan metabolism pathway in mice. Red color indicates upregulated proteins, and blue indicates downregulated proteins, either by AC or AL treatment, revealed by DIA proteome analysis. Nor: *wt*/*wt* mice treated with vehicle. Con/control: db/db mice treated with vehicle. AC/acid: db/db mice treated with 20 mg/kg bodyweight/day cinnamic acid. AL/aldehyde: db/db mice treated with 20 mg/kg bodyweight/day cinnamic aldehyde. ^*^p < 0.05, ^***^p < 0.001.

Based on transcriptomic and proteomic results, we found that both AC and AL significantly improved mitochondrial function. GSEA analysis showed a significant upregulation in oxidative phosphorylation (OXPHOS) pathway in treated group, with an enrichment score of 0.826 and 0.535 for the AC group and AL group, respectively (Figure 5A). GSEA analysis using the GO database showed the same enrichment tendency in the respiratory chain pathway (Supplementary Figure 3). For DEPs enriched in OXPHOS pathway, AC significantly upregulated 5 out of 6 (83.33%), which are NDUFA3, NDUFA7, NDUFB7, SDHD and ATP5E, and AL significantly upregulated 7 out of 11 (63.6%), which are ND2, ND3, NDUFS7, NDUFB5, NDUFB6, SDHDM and ATP6V1C1. The heatmap showed that many genes and proteins of complexes involved in OXPHOS were upregulated by treatments with AC or AL (Figure 5B). We further examined the expression of some of these proteins by PRM, and most of the results were consistent with the proteome except NDUFS4, for which the proteome data showed it was suppressed, whereas the fold changes in PRM were both above 1 (Figure 5C). In line with this evidence, we believe that AC and AL treatment may improve mitochondrial function through enhancing the OXPHOS efficacy (Figure 5D). Conversely, the ATP content of treated groups was significantly lower than that in the control group (Figure 5E), which is interesting because the OXPHOS process generates ATP in order to provide energy for life activities. We assume it was because in AC and AL groups, the ATPases were upregulated (Figures 6A,B), which consumed the generated ATP.

### Effect of AC and AL on Tryptophan Metabolism

It is reported that excess glucocorticoid may play a role in the development of insulin resistance in hepatocytes by increasing serum serotonin (44, 45), and this mechanism was proven in db/db mice (46, 47). The bioinformatic analysis suggested that the DEGs and DEPs were significantly enriched in the tryptophan metabolism pathway, AC and AL significantly increased the gene and protein expression of tryptophan metabolism. AC and AL both significantly upregulated IDO2, MAOB, ALDH7A1 and AL also significantly induced IDO1 and KYAT1 (Figures 6C,D). The majority of tryptophan is metabolized to kynurenine and eventually forms NAD^+^ (48) and contributes to the energy metabolism. According to the ELISA assay of the serotonin contents in mice livers, AC and AL both significantly reduced the serotonin content in db/db mice (Figure 6E). Taking the evidence together, both chemicals may regulate the metabolism of tryptophan, especially increasing the degradation of serotonin in the liver (Figure 6F).

### Effect of AC and AL on Autophagy Mediated Lipid Clearance

**Figure 7:**
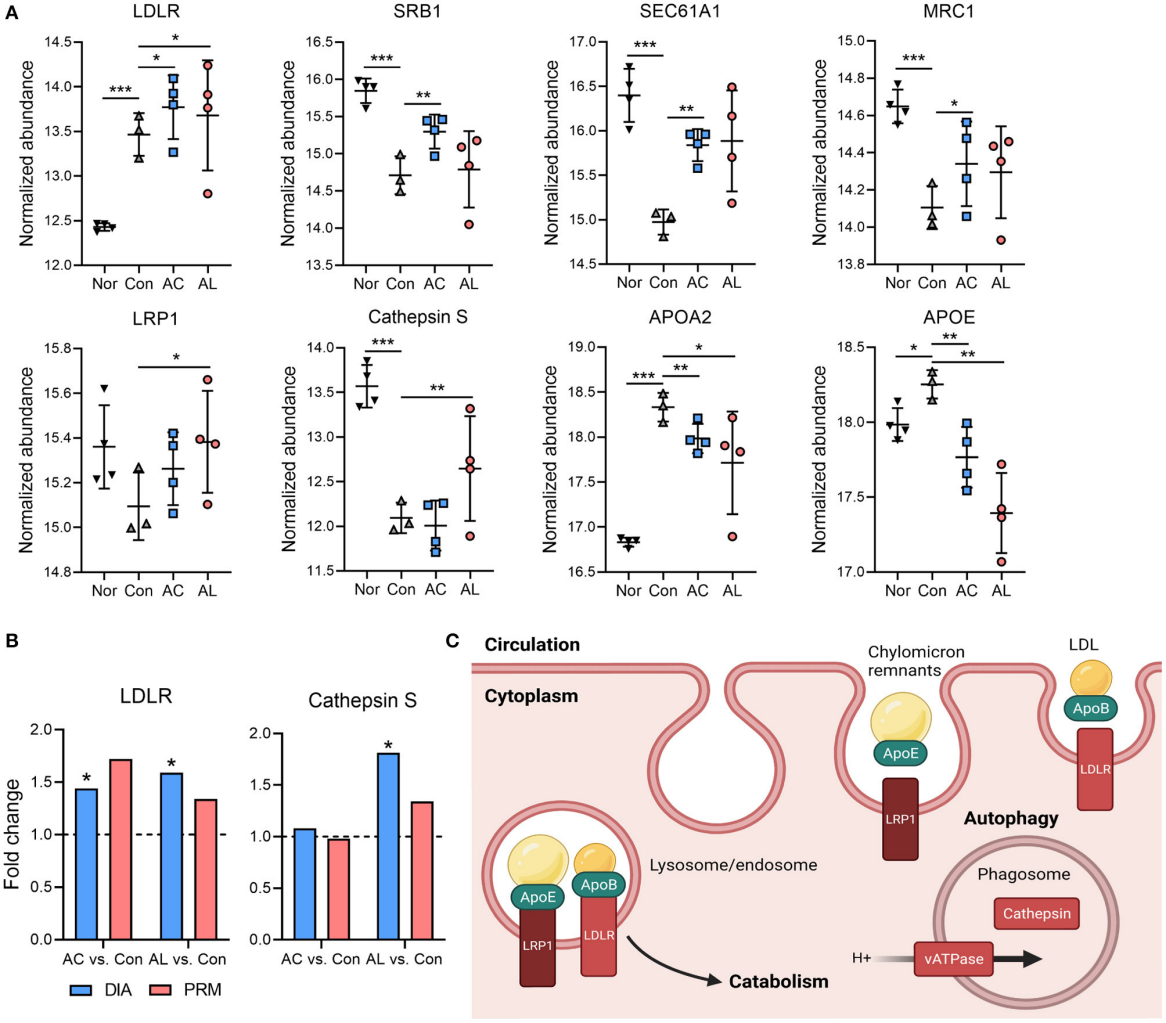
Effect of AC and AL on autophagy mediated lipid clearance. **(A)** Normalized abundance of indicated proteins in mice livers. Data are quantile normalized before log2 transformation. **(B)** Results from PRM analysis. Blue column indicates the fold change of DIA proteome analysis, and red column indicates the fold change of PRM verification. **(C)** Diagram of LRP1 and LDLR mediated lipoprotein clearance and autophagy in mice liver. Nor: *wt*/*wt* mice treated with vehicle. Con/control: db/db mice treated with vehicle. AC/acid: db/db mice treated with 20 mg/kg bodyweight/day cinnamic acid. AL/aldehyde: db/db mice treated with 20 mg/kg bodyweight/day cinnamic aldehyde. ^*^p < 0.05, ^**^p < 0.01, ^***^p < 0.001.

The autophagy process enables cells to recycle essential cytoplasmic materials and adapt to stress conditions. It is well established that autophagy declines in obese and diabetic states (49, 50). Results from omics suggested that AC and AL may affect autophagy of mice hepatocytes. A large number of DEGs were enriched in the phagosome, endocytosis and lysosome (Supplementary Table 4). In the proteome, AC significantly upregulated LDLR, SRB1, SEC61A1 and MRC1, all associated with lipid metabolism. AL significantly increased the expression of LDLR, LRP1, Cathepsin S (Figures 7A,B) and vATPase (ATP6V1C1, Figures 6A,B), which are associated with LDL clearance and the formation of phagosomes. We further confirmed the components of lipoproteins, and the results were consistent with findings above. In AC and AL groups, the content of APOA2 and APOE was significantly decreased (Figure 7A). These results indicate restored autophagy mediated hepatic lipid clearance in the treated mice (Figure 7C).

## Discussion

With the accelerating growth of the incidence rate of metabolic disorders, new strategies of preventing and treating these diseases are required. As a commonly used spice worldwide, cinnamon has been proved to possess multiple beneficial effects (16, 21, 51). Here we used combined omics to investigate two major active components in cinnamon, AC and AL, and their effects on db/db mice. Db/db is a stable and efficient genetic model for metabolic diseases and mimics the pathological changes in human including insulin resistance, disrupted glucolipid metabolism and excessive lipid accumulation (52).

In the current study, AC and AL were administered to db/db mice by gavage at the dosage of 20 mg/kg bodyweight/day for 4 weeks. *Wt*/*wt* mice were used as a normal group, and db/db mice were used as a control group. No significant change of body length or femur length was detected in the treated group. The treatments also did not significantly affect the weight of heart, spleen, or brain of mice. Compared with normal group, mice in control group showed enlarged kidney, which probably due to swelling caused by diabetic nephropathy. AL treatment attenuated the increased kidney weight of db/db mice (Supplementary Table 5). Along with the food intake results, these data suggested the treatments did not cause deleterious health effects in db/db mice and did not affect the lean mass of mice. Compared with *wt*/*wt* mice, the db/db mice displayed elevated body weight and blood glucose levels throughout the intervention. The bodyweight and blood glucose of mice showed no significant difference between the control group and treated groups for the first two weeks, but at the end of the treatment the bodyweight and blood glucose of both AC and AL groups were significantly lower compared with those of db/db mice in the control group. Meanwhile, the ITT experiment at the fourth week demonstrated that AC and AL significantly boosted insulin sensitivity in db/db mice. These results indicate that AC or AL may not be suitable for use as acute hypoglycemic treatments but are effective long term.

AC and AL showed beneficial effects on multiple parameters involved in metabolism. As a display of perturbed energy metabolism, db/db mice also showed markedly increased liver mass due to hepatic lipid accumulation. As indicated by histological observation and biochemical assessment, the treatments partially restored the size of lipid droplets in mice liver and improved serum lipid profile. Although no impact was observed in TC or LDL levels, the chemicals exhibited significant hypolipidemic effect on TG and FFA levels. Furthermore, 20 mg/kg bodyweight/day of AC and AL did not affect liver and renal function of mice, consistent with previous studies of their toxicity (26, 29, 53). As these chemicals were proved to be effective, we further explored the potential mechanisms underlying their effects using transcriptome and proteome analysis. The experiments were performed on mice livers as the chemicals significantly reduced the liver mass and liver index, instead of the weight of adipose tissues, in treated groups compared with the control. It is also worth noting that a large portion of AL is metabolized into AC and cinnamyl alcohol in the body (31), and probably due to its direct utilization, AC was previously demonstrated to have greater hypoglycemic effects than AL (27). This may also be the reason for the similar mechanisms we identified in this study.

Despite the definite hypoglycemic and hypolipidemic effects suggested by the mice phenotype, AC and AL did not show a strong impact on the translational expression of enzymes regulating glucolipid metabolism. Some proteins showed a tendency to change, though few were significant. According to the DIA results, AC treatment significantly induced the protein level of GLUT2, which is reported to mediate the glucose transportation between liver and plasma (54), the depletion of which causes suppressed hepatic glucose uptake and hyperglycemia (55, 56). However, the increased glucose uptake by upregulated GLUT2 protein may be partially blunted by the upregulation of PEPCK, a rate-limiting enzyme in the gluconeogenesis process (57). As the changes in glucolipid metabolism enzymes were not significant, we assumed other possible mechanisms underlying the beneficial effects of these chemicals that may provide explanations for the improvements in phenotypes of mice in treated groups compared with the control group. Thus, we performed functional enrichment analyses on DEGs and DEPs based on KEGG and GO databases.

Previous studies have highlighted the link between degradation in mitochondrial function in skeletal muscles with the development of metabolic disorders including insulin resistance, indicated by decreased mitochondrial biogenesis and respiration process (58, 59). As for the liver, it usually shows elevated lipid content called non-alcoholic fatty liver disease (NAFLD) and progresses to non-alcoholic steatohepatitis (NASH) with the aggregation of metabolic disorders. As an adaptive change to the increased lipid accumulation, mitochondrial respiration enhances in the NAFLD stage to respond to energy substrates overloading. However, the maximal mitochondrial respiration decreases in the NASH stage, suggesting the adaptive ability of mitochondria has crossed its threshold, and the mitochondrial function starts to decline (60–62). The functional enrichment analysis showed a profound enrichment in genes and proteins relating to mitochondria, the mitochondrial membrane and mitochondrial respiratory complexes. The OXPHOS pathway was significantly increased in treated groups compared with the control group, indicating higher OXPHOS flux in the treated groups. Taking the decreased lipid droplets observed in H&E staining into consideration, the increased OXPHOS protein expression could either suggest an upregulated adaptive threshold or a prevention of aggregation of hepatic pathological changes, implying a restored mitochondrial function in hepatocytes. Since the OXPHOS process transfers electrons and forms ATP for driving cellular biofunctions, and almost all components involved in OXPHOS were upregulated, the ATP generation would be increased in mice in the treated groups. However, the ATP content assessment was not consistent with the upregulated gene and protein expression in the mitochondrial respiration pathway. We thus examined the ATP consumption in the liver, represented by the expression of ATPases.

As expected, AC significantly increased the expression of ATP1B1, and AL significantly increased ATP1B1, ATP2B1, and ATP6V1C1. ATP1B1 is the Na^+^/K^+^-ATPase β 1 subunit, which is critical for the maintenance of membrane voltage potential (63). ATP2B1 is the Ca^+^-transporting ATPase and maintains the intracellular calcium homeostasis, and systematic downregulation of the encoding gene leads to significantly elevated blood pressure (64, 65). ATP6V1C1 is the C1 subunit of vacuolar-ATPase, and it pumps H^+^ into phagosomes or lysosomes, thus ensuring the acidity of these cellular compartments and thus their functionality (66). All these results might provide an explanation for the inconsistency found in the significantly upregulated mitochondrial respiratory chain and the unexpected decrease in hepatic ATP content in mice in treated groups. It was previously reported that Na^+^/K^+^-ATPase is highly relevant in the regulation of cardiovascular complications (67), and the activity of hepatic Na^+^/K^+^-ATPase declines in metabolic disorders including obesity and diabetes (68, 69). Thus, induction of ATP1B1 may suggest another beneficial effect of AC and AL on db/db mice.

The results of KEGG enrichment of DEGs suggested alterations in amino acid metabolism in AC and AL groups. Since the amino acid metabolism is highly associated with obesity and insulin resistance (70), we further investigated the effects of AC and AL on amino acid metabolic pathways. Of note, nearly all DEPs enriched in tryptophan metabolism pathways were upregulated after treatments, and the content of serotonin, an intermediate in the metabolism of tryptophan, showed a significant decrease in treated groups. Serotonin is considered to induce lipid accumulation in both humans and animals, and suppression of its generation in mice led to a significantly lower body weight (71). Increased hepatic serotonin content impairs insulin sensitivity in a mechanistic target of rapamycin (mTOR)-dependent manner (45). AC and AL significantly enhanced the catabolism of serotonin and decreased its content, which could subsequently ameliorate insulin resistance in db/db mice.

We found other possible mechanisms for the effects of AC and AL as well. In hepatocytes, LDLR and LRP1 bind to their ligands, namely apolipoproteins (Apos), to assert their lipid-clearance functions by internalizing the lipoproteins to endo- or lysosomes (72, 73), which are the endpoints of autophagy and endocytosis (74). Autophagy is impaired during metabolic disorders (49), and autophagy deficiency in mice led to obesity-related metabolomic profile changes (75). Analysis of the proteome showed that AC significantly upregulated LDLR and AL significantly upregulated LDLR and LRP1, which mediated the clearance of TG- and cholesterol ester-containing lipoproteins including LDL and chylomicrons (76, 77). LRP1 inactivation in LDLR-deficient background mice dramatically suppressed the clearance of serum lipids because of a decline in the endocytosis rate and reduction of lipase activity (76, 78). Interestingly, an *in vitro* study demonstrated the negative regulatory effect of liver X receptor (LXR) on LDLR expression (79), and our previous study showed AC treatment in db/db mice downregulated the mRNA expression of LXR (43), which might partially contribute to the upregulation of LDLR proteins. AC significantly upregulated SEC61A1, the mutation of which was reported to cause ER stress in hepatocytes, and the mutant mice displayed a diabetic phenotype (80). AL significantly increased Cathepsin S, which was demonstrated to decrease in impaired hepatic lipid metabolic situations as a lysosomal enzyme (81). Along with the aforementioned ATP6V1C1, which assures the acidity environment inside lysosomes, it is deducible that the treatments improved the autophagy in hepatocytes and contributed to lipid metabolism. This is further supported by the significantly decreased TG content and downregulated Apos expression.

There are discrepancies identified between two omics, indicating different transcriptional and translational regulations in proteins (Supplementary Figure 1). We assume this may be attributed to two reasons: Firstly, our protein-extraction approach did not isolate the membrane, thus may cause an underrepresentation of membrane-associated proteins in the DIA results. It should be acknowledged that in terms of representing the entire set of genes expressed in cells, the transcriptomic results seem to be more accurate. Secondly, a large portion of proteins go through post-transcriptional regulation and this could also lead to inconsistencies between the mRNA levels and protein expressions. The expression of ribosomal proteins was largely altered in both treated groups, as indicated by the KEGG enrichment analysis (Supplementary Figure 5). In addition, qPCR analysis was used to verify the accuracy of transcriptome, similar tendencies were observed between transcriptomic and PCR results (Supplementary Figures 6a,b). PRM were employed to verify the protein expression, and a strong correlation was found between the DIA results and PRM results (Supplementary Figure 6c), suggesting the DIA proteomic results were relatively robust and trustworthy.

In the current study, we focused on the molecular signaling in the liver after AC or AL treatments. However, metabolic diseases are usually systematic and involve multiple organs and systems, and future studies may investigate the effects of these chemicals on other tissues. Moreover, although db/db is a commonly used animal model for metabolic diseases, the mechanisms of murine models can be different from those of patients. Thus, these mechanisms should be carefully verified by more experiments on different models before being adopted in human studies.

## Conclusion

To summarize, after 4 weeks of treatment, both AC and AL ameliorated the metabolic disorders in db/db mice. The transcriptomics and proteomics identified several possible mechanisms for their therapeutic effects, probably due to their metabolic characteristics in the body, AC and AL share very molecular mechanisms: (1) improving the mitochondrial function, especially improving the OXPHOS process; (2) increasing tryptophan metabolism and decreasing serotonin levels and (3) improving autophagy and accelerating TG- and cholesterol-containing lipid clearance. Considering their low toxicity, we propose that AC and AL are promising candidates for the prevention and treatment of metabolic disorders.

## Data Availability Statement

The datasets presented in this study can be found in online repositories. The raw reads of transcriptomic data are deposited in Sequence Read Archive (SRA) database (Bioproject: PRJNA776675) and the mass spectrometry proteomics data are deposited in the ProteomeXchange Consortium (http://proteomecentral.proteomexchange.org) *via* the iProX partner repository with the dataset identifier: PXD029385.

## Ethics Statement

The animal study was reviewed and approved by Animal Care and Ethics Committee of Beijing University of Traditional Chinese Medicine.

## Author Contributions

YW and M-hW: conceptualization and methodology. L-lQ, L-lW, and T-hL: validation. YW, M-hW, TY, T-yQ, Y-mH, C-fZ, B-jS, and LD: investigation. YW: writing—original draft preparation. YW and TY: writing—review and editing and visualization. L-lW and T-hL: supervision and funding acquisition. All authors have read and agreed to the published version of the manuscript.

## Funding

This research was funded by the International Cooperation Research Center for the Prevention and Treatment of Diabetes by Traditional Chinese Medicine, Grant Number: 2015B01022.

## Conflict of Interest

The authors declare that the research was conducted in the absence of any commercial or financial relationships that could be construed as a potential conflict of interest.

## Publisher's Note

All claims expressed in this article are solely those of the authors and do not necessarily represent those of their affiliated organizations, or those of the publisher, the editors and the reviewers. Any product that may be evaluated in this article, or claim that may be made by its manufacturer, is not guaranteed or endorsed by the publisher.
