# Supplementary material for: Mechanisms for Improving Hepatic Glucolipid Metabolism by Cinnamic Acid and Cinnamic Aldehyde: An Insight Provided by Multi-Omics
Source: Front Nutr. 2022 Jan 11;8:794841. doi: 10.3389/fnut.2021.794841 (PMC8786797; doi:10.3389/fnut.2021.794841)
Supplement: Supplementary file 1 [file Data_Sheet_1.docx]

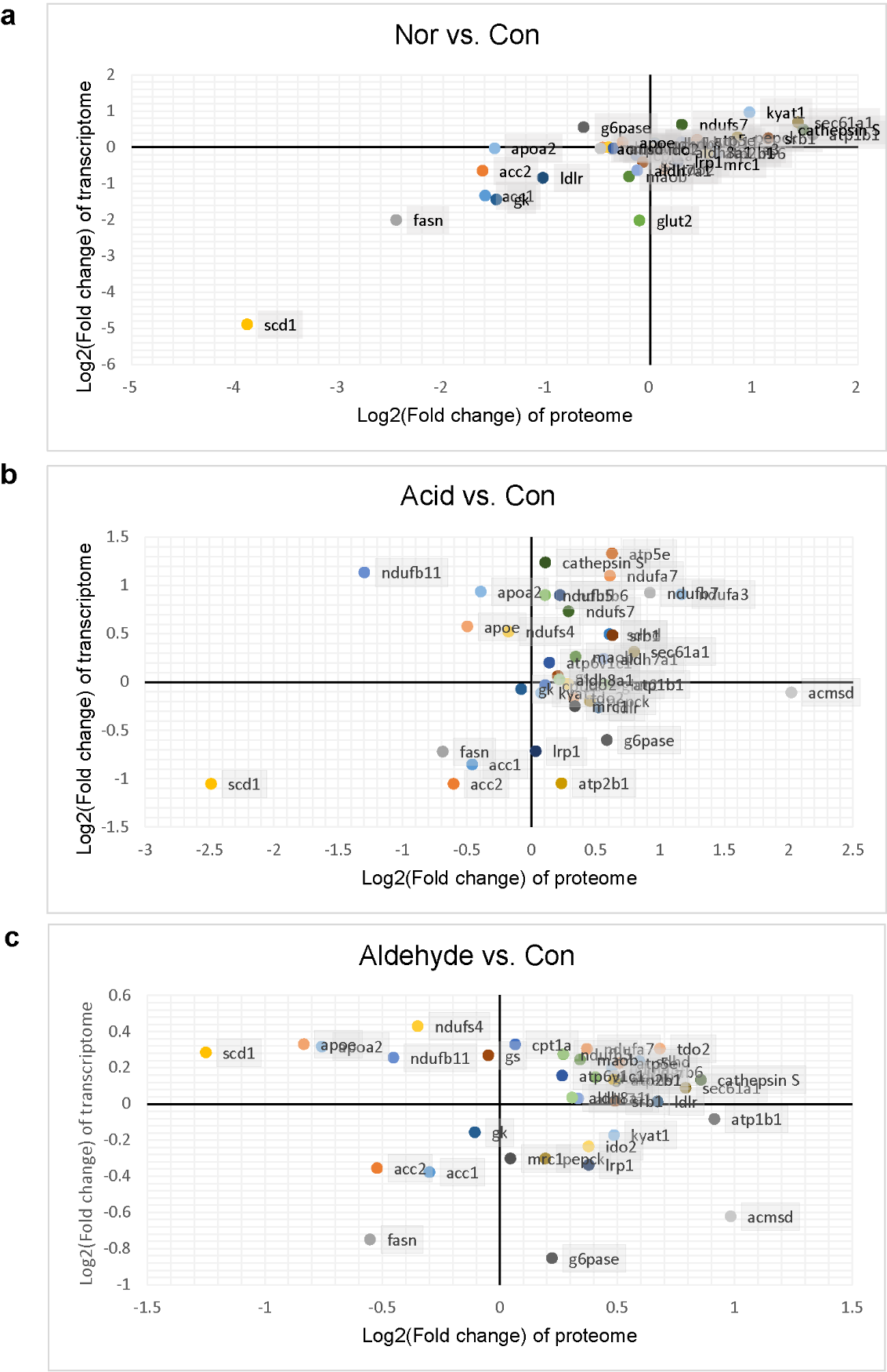


**Supplementary Figure 1.** Relationship between mRNA expression and protein expression of identified key factors visualized by four-quadrant diagram.

**
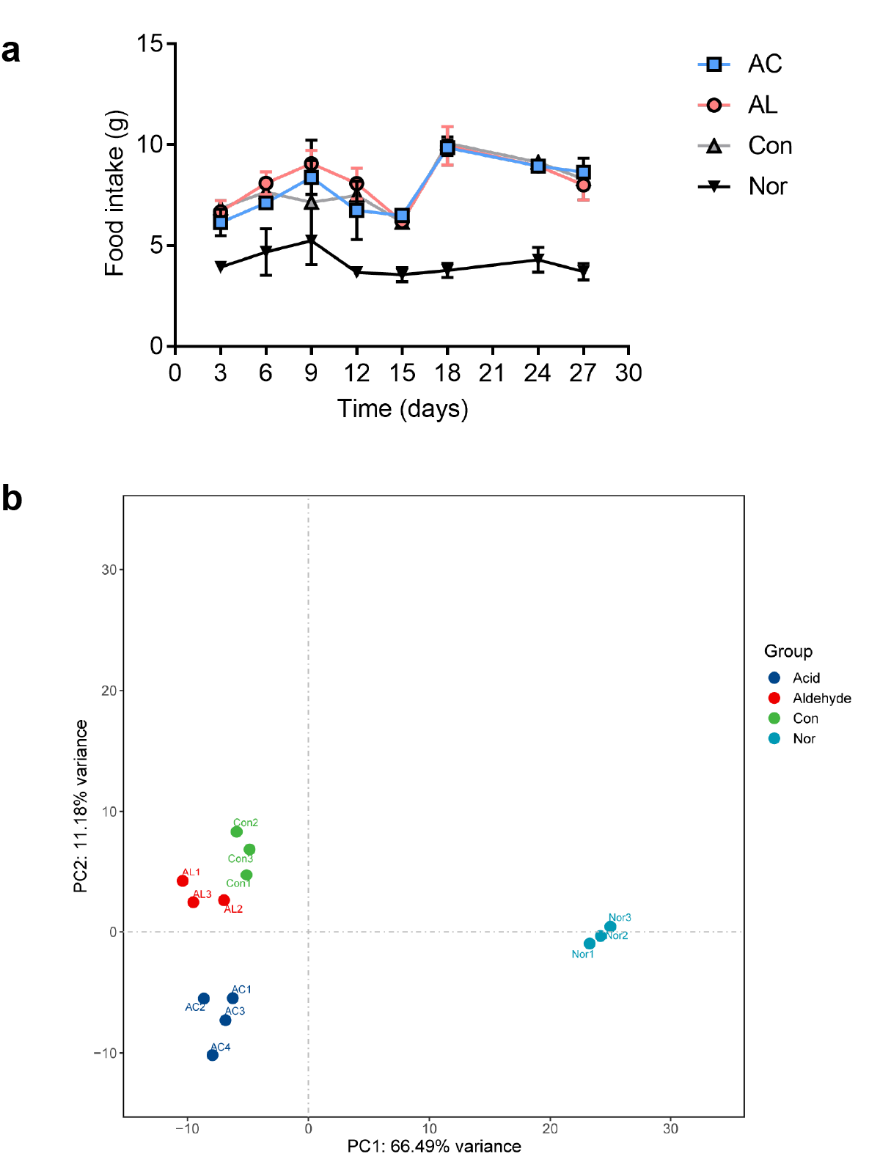
**

**Supplementary Figure 2.** **(a)** Food intake of mice. **(b)** PCA analysis of transcriptomic results.

**
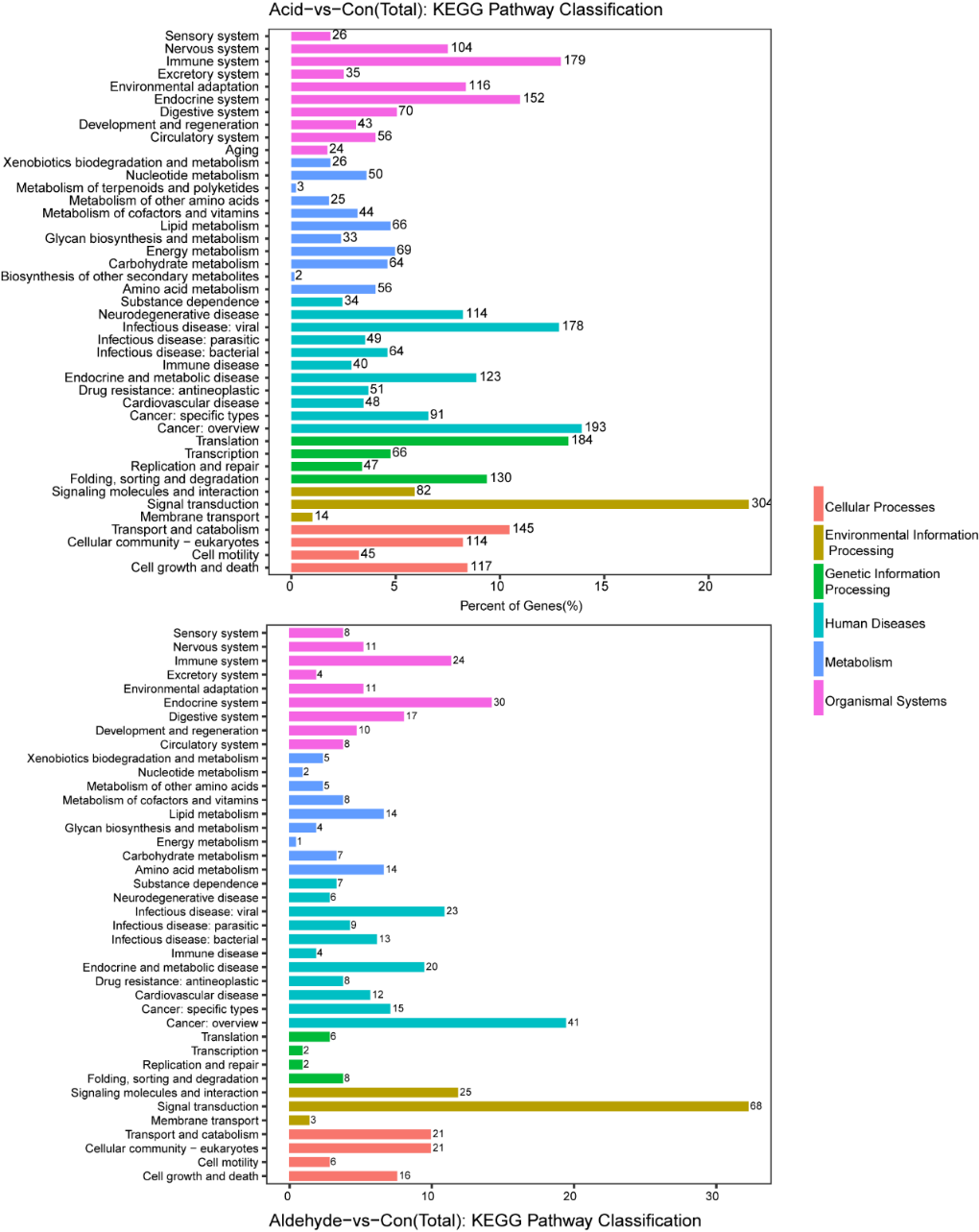
**

**Supplementary Figure 3.** KEGG enrichment of DEGs of Acid vs Control (up) and Aldehyde vs Control (down).

**
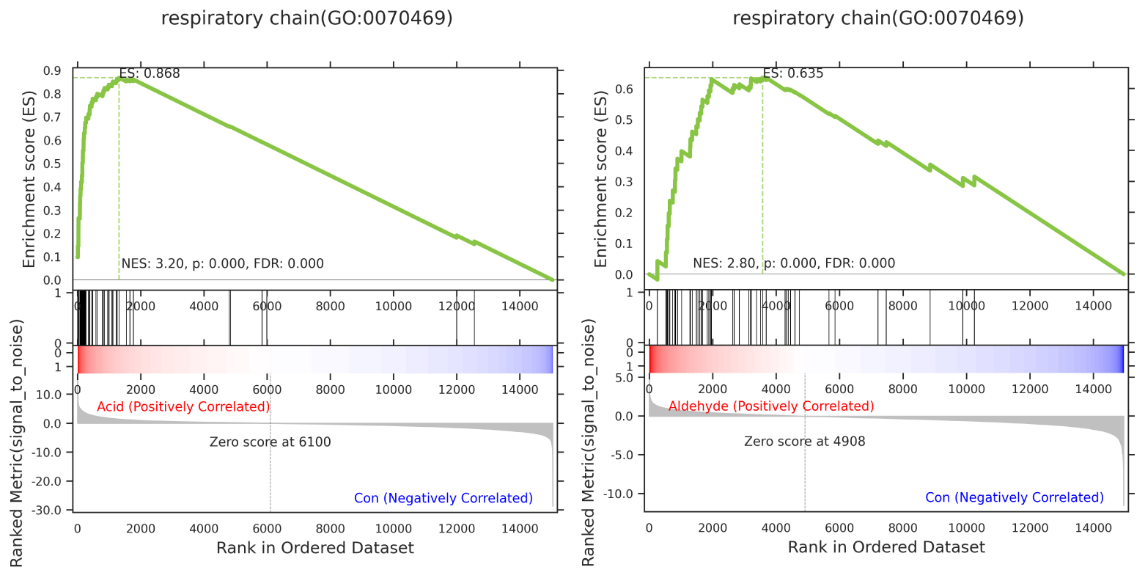
**

**Supplementary Figure 4.** AC and AL significantly upregulated genes in GO: 0070469 respiratory chain pathway.


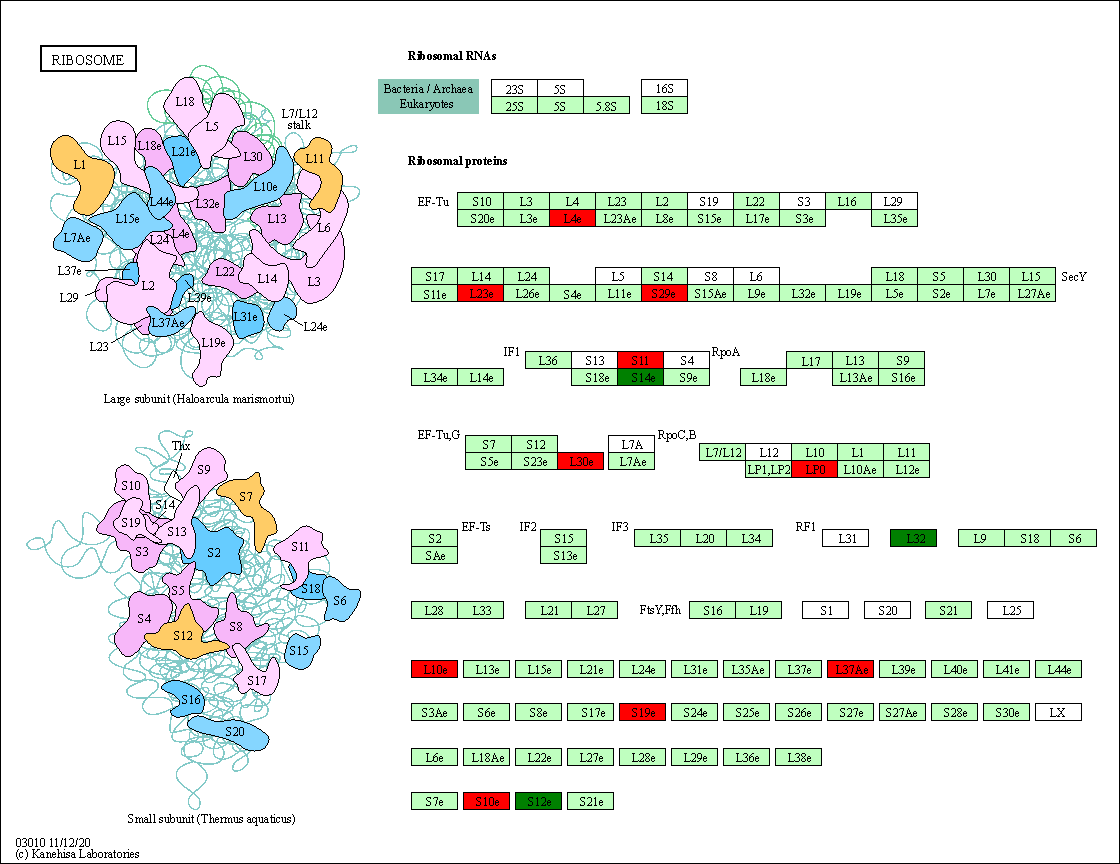


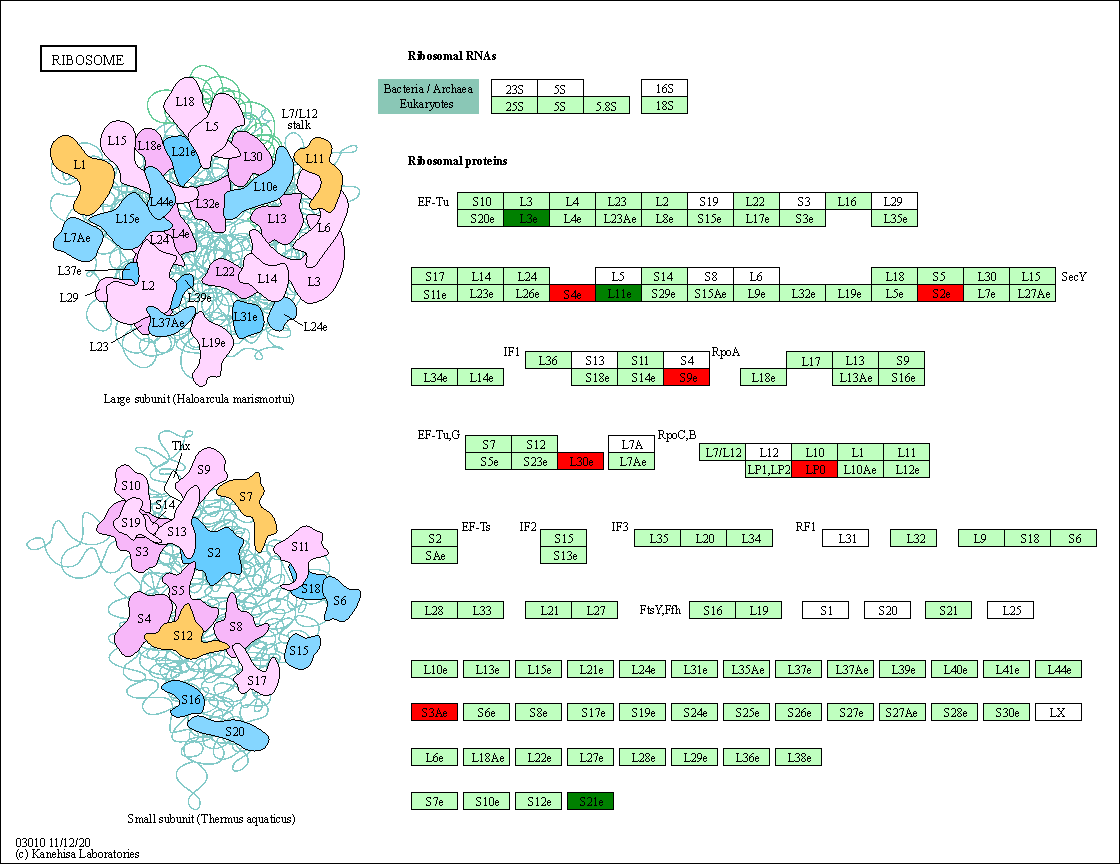


**Supplementary Figure 5.** DEPs of Acid vs Control (up) and Aldehyde vs Control (down) enriched in KEGG pathway mmu03010: ribosome.


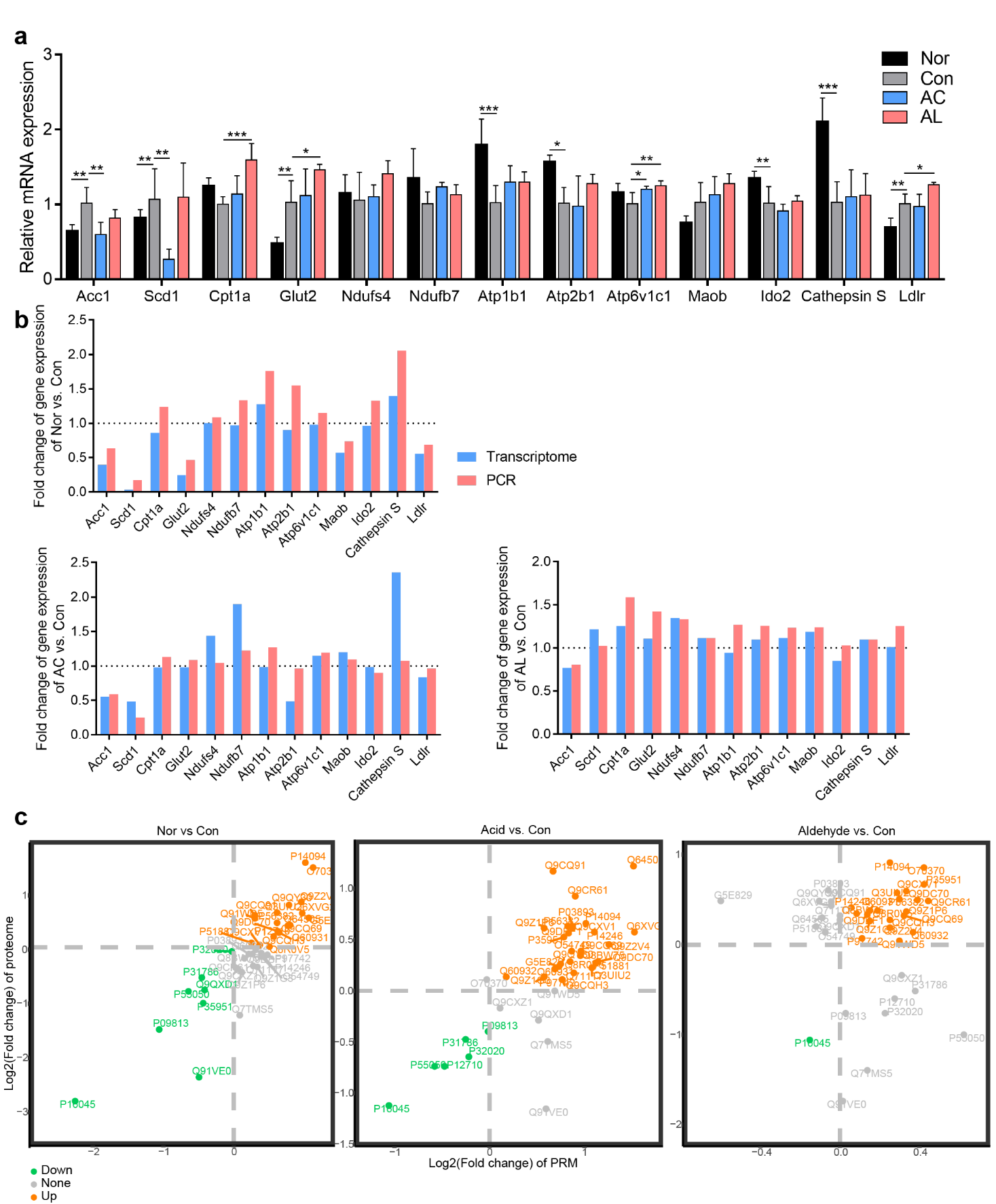


**Supplementary Figure 6.** Verification of transcriptome and DIA results. (**a**) Results of qPCR analysis (one-way ANOVA analysis applied, *N*=6/group). (**b**) Fold change of genes from transcriptome and qPCR. (**c**) Fold change of verified proteins in DIA and PRM.

| Genes | Forward primer (5’→3’) | Reverse primer (5’→3’) |
| --- | --- | --- |
| Acc1 | GAATCTGGCTGCATCCATTAT | AAGCTCAGCCTGTTGAACTTTA |
| Scd1 | TCTTCCCAGAGACTGACAGATA | GGGAAAGGTTTCCTGCAAT |
| Cpt1a | GATGTTTCGACAGGTGGTT | CAGGAATGCTCTGCGTTTAT |
| Glut2 | TTGTGCTGCTGGATAAATTCG | GACCTGGCCCAATCTCAA |
| Ndufb7 | CTGAAGTGCAAGCGAGAC | CTCACGTTCAAACTCCTTCAT |
| Ndufs4 | CTCGCAATAACATGCAGTCT | TGCCCAACCCATCAAAGG |
| Atp1b1 | TGGCTGGCATCTTCATCG | CACTCGGTCCTGGTATGT |
| Atp2b1 | CCAACAACTCAGTTGCGTAT | CTGTAAGCGTAATTCCAAAGTC |
| Atp6v1c1 | GCAGTTGATGATTTCAGACACA | TGCTTTCATCTCCTCCTCG |
| Maob | CCATGAGGTCTCTGCACTA | CCATTGGTTGTTGAGATGATTC |
| Ido2 | CAGTAACCTGGAAACCATCA | TCTCCACCAAGACTGTCACTA |
| Cathepsin S | AAAGCGGTGTCTATGACG | ATCTTTGCCATCAAGAGTCC |
| Ldlr | TGCTCCCAGGATGACTTC | ATCTAGGCAATCTCGGTCT |
| β-actin | GGCTCCTAGCACCATGAAGA | AGCTCAGTAACAGTCCGCC |

**Supplementary Table 1.** Primers used in qPCR analysis.

| Comparisons | Term rank | Term | ES | NES | *p*-value | FDR |
| --- | --- | --- | --- | --- | --- | --- |
| Acid vs. Con KEGG | 1 | Ribosome(mmu03010) | 0.864186 | 3.823349 | 0 | 0 |
|  | 2 | Oxidative phosphorylation(mmu00190) | 0.825994 | 3.472092 | 0 | 0 |
|  | 3 | Parkinson's disease(mmu05012) | 0.807529 | 3.400807 | 0 | 0 |
|  | 4 | Huntington's disease(mmu05016) | 0.679113 | 3.053417 | 0 | 0 |
|  | 5 | Alzheimer's disease(mmu05010) | 0.680342 | 3.015357 | 0 | 0 |
|  | 6 | Thermogenesis(mmu04714) | 0.602615 | 2.729828 | 0 | 0 |
|  | 7 | Non-alcoholic fatty liver disease (NAFLD)(mmu04932) | 0.643573 | 2.716021 | 0 | 0 |
|  | 8 | Retrograde endocannabinoid signaling(mmu04723) | 0.621033 | 2.560506 | 0 | 0 |
|  | 9 | Proteasome(mmu03050) | 0.681678 | 2.445623 | 0 | 0 |
|  | 10 | Cardiac muscle contraction(mmu04260) | 0.650346 | 2.414564 | 0 | 0 |
| Acid vs. Con GO | 1 | structural constituent of ribosome(GO:0003735) | 0.850905 | 3.844853 | 0 | 0 |
|  | 2 | ribosome(GO:0005840) | 0.790604 | 3.536152 | 0 | 0 |
|  | 3 | respiratory chain(GO:0070469) | 0.867767 | 3.204154 | 0 | 0 |
|  | 4 | mitochondrial respiratory chain complex I(GO:0005747) | 0.872026 | 3.137609 | 0 | 0 |
|  | 5 | cytosolic large ribosomal subunit(GO:0022625) | 0.829618 | 3.127139 | 0 | 0 |
|  | 6 | translation(GO:0006412) | 0.652701 | 3.057655 | 0 | 0 |
|  | 7 | mitochondrial large ribosomal subunit(GO:0005762) | 0.814776 | 2.957408 | 0 | 0 |
|  | 8 | cytoplasmic translation(GO:0002181) | 0.822924 | 2.874507 | 0 | 0 |
|  | 9 | mitochondrial respiratory chain complex I assembly(GO:0032981) | 0.80095 | 2.835631 | 0 | 0 |
|  | 10 | mitochondrial inner membrane(GO:0005743) | 0.596517 | 2.798066 | 0 | 0 |
| Aldehyde vs. Con KEGG | 1 | Ribosome(mmu03010) | 0.762636 | 3.958534 | 0 | 0 |
|  | 2 | Oxidative phosphorylation(mmu00190) | 0.534629 | 2.569553 | 0 | 0 |
|  | 3 | Parkinson's disease(mmu05012) | 0.51491 | 2.396979 | 0 | 0 |
|  | 4 | Fatty acid degradation(mmu00071) | 0.511509 | 2.297476 | 0 | 0.000986 |
|  | 5 | Proteasome(mmu03050) | 0.530762 | 2.246627 | 0 | 0.000822 |
|  | 6 | Biosynthesis of unsaturated fatty acids(mmu01040) | 0.590021 | 2.211992 | 0 | 0.001409 |
|  | 7 | Peroxisome(mmu04146) | 0.420469 | 2.06347 | 0 | 0.001233 |
|  | 8 | Fatty acid elongation(mmu00062) | 0.512186 | 1.995037 | 0 | 0.003836 |
|  | 9 | Cardiac muscle contraction(mmu04260) | 0.437228 | 1.96642 | 0 | 0.003946 |
|  | 10 | Alzheimer's disease(mmu05010) | 0.333553 | 1.740986 | 0 | 0.02511 |
| Aldehyde vs. Con GO | 1 | cytosolic large ribosomal subunit(GO:0022625) | 0.76459 | 3.513912 | 0 | 0 |
|  | 2 | structural constituent of ribosome(GO:0003735) | 0.730695 | 3.496853 | 0 | 0 |
|  | 3 | cytosolic small ribosomal subunit(GO:0022627) | 0.713141 | 3.114977 | 0 | 0 |
|  | 4 | cytoplasmic translation(GO:0002181) | 0.687936 | 2.985266 | 0 | 0 |
|  | 5 | respiratory chain(GO:0070469) | 0.635271 | 2.800446 | 0 | 0 |
|  | 6 | mitochondrial large ribosomal subunit(GO:0005762) | 0.629688 | 2.779915 | 0 | 0 |
|  | 7 | large ribosomal subunit(GO:0015934) | 0.836039 | 2.749677 | 0 | 0 |
|  | 8 | threonine-type endopeptidase activity(GO:0004298) | 0.770091 | 2.713769 | 0 | 0 |
|  | 9 | proteasome core complex(GO:0005839) | 0.762544 | 2.568992 | 0 | 0 |
|  | 10 | mitochondrial respiratory chain complex I(GO:0005747) | 0.616856 | 2.565199 | 0 | 0 |

**Supplementary Table 2.** Top 10 significantly enriched KEGG pathways and GO terms from GSEA analysis.

| Proteins | Modified Petite Sequence |
| --- | --- |
| CPT1A | _EVLSEPWR_  _LAALTAADR_  _AGNTIHAILLYR_ |
| GLUT2 | _VSVIQLFTDANYR_  _FGPAHALIIAGR_  _HVLGVPLDDR_ |
| PEPCK | _ENALNLK_  _FLWPGFGENSR_  _YLAAAFPSAC[Carbamidomethyl (C)]GK_ |
| NDUFA7 | _AVTPAPPMK_  _EVVPPSIIMSSQK_  _LSNNYYC[Carbamidomethyl (C)]TR_ |
| NDUFB7 | _DYC[Carbamidomethyl (C)]AHYLIR_  _DSFPNFLAC[Carbamidomethyl (C)]K_ |
| NDUFS4 | _LDITTLTGVPEEHIK_  _SYGANFSWNK_ |
| SDHD | _AASLHWTSER_  _AVAMLWK_ |
| UQCRQ | _HVISYSLSPFEQR_  _EFGNLAR_ |
| ATP5E | _QAGLSYIR_  _FSQIC[Carbamidomethyl (C)]AK_ |
| ATP1B1 | _VAPPGLTQIPQIQK_  _YNPNVLPVQC[Carbamidomethyl (C)]TGK_  _SYEAYVLNIIR_ |
| ATP2B1 | _IQESYGDVYGIC[Carbamidomethyl (C)]TK_ |
| ATP6V1C1 | _QFGPLVR_  _QYETLAEMVVPR_  _SSNVLSEDQDSYLC[Carbamidomethyl (C)]NVTLFR_ |
| MAOB | _YVDLGGSYVGPTQNR_  _VLNSQEALQPVHYEEK_ |
| IDO2 | _ALVQGMEAIR_  _IFLSGWK_ |
| LDLR | _GVSSPC[Carbamidomethyl (C)]SSLEFHC[Carbamidomethyl (C)]GSSEC[Carbamidomethyl (C)]IHR_  _NVVALDTEVTNNR_  _NINSINFDNPVYQK_ |
| Cathepsin S | _NHC[Carbamidomethyl (C)]GIASYC[Carbamidomethyl (C)]SYPEI_  _YIQLPFGDEDALK_ |

**Supplementary Table 3.** Unique peptides of proteins for PRM verification.

| Comparison | Upregulated genes | Downregulated genes |
| --- | --- | --- |
| AC vs Con | Gm10499, Gm11127, H2-T22, H2-T23, Rab5c, Atp6v0b, Atp6v0c, Atp6v0d1, Atp6v0e, Atp6v1e1, Atp6v1f, Atp6v1g1, Cd14, Colec11, Cyba, Marco, Sec61b, Sec61g, Tlr2, Clta, Cltb, Ap2s1, Arf1, Arf5, Arpc1b, Arpc3, Arpc4, Arpc5l, Capzb, Chmp1a, Chmp2a, Chmp6, Hras, Hspa2, Hspa8, Mvb12a, Pard6a, Rab4a, Snf8, Snx3, Ubb, Vps25, Vps28, Vps29, Acp5, Ap1s1, Cd63, Ctsh, Dnase2a, Fuca1, Laptm4a, Lgmn, Litaf, Npc2, Pla2g15 | Eea1, Colec12, Dync1h1, Dync1li2, Dync2h1, Itga2, Itga5, Itgav, Itgb3, Pikfyve, Pla2r1, Sec61a2, Sftpa1, Tubb2a, Igf2r, Acap2, Arap2, Arfgef1, Asap2, Fgfr3, Fgfr4, Kif5a, Kif5b, Mdm2, Nedd4l, Psd3, Rab11fip2, Rabep1, Tgfbr1, Vps37a, Wwp1, Zfyve16, Abca2, Ap1g2, Ap3m1, Ap4e1, Gga3, Hyal1, Idua, Mfsd8 |
| AL vs Con | H2-DMb2, H2-Ea-ps, Gm11127, Cd63, Ctse | Itga5, Itgb3, Sec61a2, Sftpa1, Tubb2a, Arap3, Arrb1, Asap2, Grk5, Kif5a, Nedd4l, Snx32, Gga3 |

**Supplementary Table 4.** Differentially expressed genes enriched in KEGG lysosome (mmu04142), endocytosis (mmu04144) and phagosome (mmu04145) pathways.

| Group | Body length (mm) | Femur length (mm) | Heart weight (g) | Spleen weight (g) | Kidney weight (g) | Brain weight  (g) |
| --- | --- | --- | --- | --- | --- | --- |
| Nor | 157.286±1.254 | 14.034±0.45 | 0.156±0.022* | 0.088±0.013** | 0.168±0.018** | 0.43±0.018*** |
| Con | 155.714±3.147 | 13.357±0.765 | 0.136±0.012 | 0.056±0.011 | 0.191±0.007 | 0.372±0.014 |
| AC | 153±1.633 | 13.199±0.491 | 0.134±0.010 | 0.05±0.007 | 0.18±0.008 | 0.371±0.014 |
| AL | 155.857±2.41 | 12.59±1.242 | 0.133±0.02 | 0.047±0.005 | 0.164±0.015*** | 0.371±0.023 |

**Supplementary Table 5.** AC and AL showed no significant impact on the growth and development of db/db mice. *: p<0.05, **: p<0.01, ***: p<0.001, compared with control group (One-way ANOVA analysis applied for statistical analysis, *N* = 7/group).
